# Ferroptosis: Mechanism and potential applications in cervical cancer

**DOI:** 10.3389/fmolb.2023.1164398

**Published:** 2023-03-21

**Authors:** Xiangyu Chang, Jinwei Miao

**Affiliations:** Department of Gynecologic Oncology, Beijing Obstetrics and Gynecology Hospital, Capital Medical University, Beijing, China

**Keywords:** cervical cancer, ferroptosis, lipid peroxidation, iron accumulation, treatment

## Abstract

Ferroptosis is a distinct form of cell death mechanism different from the traditional ones. Ferroptosis is characterized biochemically by lipid peroxidation, iron accumulation, and glutathione deficiency. It has already demonstrated significant promise in antitumor therapy. Cervical cancer (CC) progression is closely linked to iron regulation and oxidative stress. Existing research has investigated the role of ferroptosis in CC. Ferroptosis could open up a new avenue of research for treating CC. This review will describe the factors and pathways and the research basis of ferroptosis, which is closely related to CC. Furthermore, the review may provide potential future directions for CC research, and we believe that more studies concerning the therapeutic implications of ferroptosis in CC will come to notice.

## Introduction

Cervical cancer (CC) is one of the most common gynecological tumors worldwide, and 311,000 women died of cervical cancer in 2018 (Bray et al., 2018). Based on the clinical stage and pathological risk factors, surgery or a combination of chemotherapy and radiation therapy is commonly used to treat CC (Koh et al., 2019). Patients with advanced-stage CC have a poor prognosis with a low 5-year mortality rate of only 17% (Pfaendler and Tewari, 2016). However, cisplatin-based first-line chemotherapy has shown little or no response (Monk et al., 2009). Significant adverse effects and narrow therapeutic windows limit the use of systemic chemotherapy. The FDA has approved atezolizumab, pembrolizumab, bleomycin sulfate, and topotecan hydrochloric acid for patients with metastatic or recurrent CC. The standard first-line therapy for CC is platinum-based chemotherapy with bevacizumab (Rallis et al., 2021); non-etheless, the failure of this treatment indicates a high likelihood of subsequent treatment failure. Therefore, it is necessary to develop novel therapeutics for the treatment of CC.

High-risk human papillomavirus (hrHPV) infection, age, smoking, and childbirth are important risk factors for CC (Gaffney et al., 2018). Among all these risk factors, persistent hrHPV infection appears to be the main risk factor responsible for the carcinogenesis and progression of CC (Crosbie et al., 2013). HPV16 is the most common HPV subtype found in CC, causing more than 50% of all cervical cancer cases (Jiang et al., 2018). hrHPV can cause cancer by expressing multiple oncoproteins, including E6 and E7 proteins, which induce carcinogenesis and malignant transformation of cervical epithelial cells (Taghizadeh et al., 2019). HrHPV infiltrates the cervical epithelium and integrates into the host genome, inactivating tumor suppressor genes and activating oncogenes. HPV oncoproteins E6 and E7 regulate the function of several tumor-related proteins, including EGFR family members, p53, and retinoblastoma protein (pRb). HPV oncoproteins also affect the function of various cellular organelles, including mitochondria (Cruz-Gregorio et al., 2019; Cruz-Gregorio et al., 2020). A study has found that HPV infection can induce chronic oxidative stress by promoting the production of reactive oxygen species (ROS) in the tissue microenvironment in patients with CC (Banerjee et al., 2019), which regulates different cellular signaling pathways (Yeo-Teh et al., 2018; Cruz-Gregorio and Aranda-Rivera, 2021) and cellular processes, such as autophagy (Aranda-Rivera et al., 2021) and apoptosis (Jiang and Yue, 2014). The concept of ferroptosis has been recently introduced as an iron-dependent form of oxidative cell death, and ferroptosis is distinct from other known forms of cell death modalities. Ferroptosis primarily occurs in cancer cells and neurons and contributes to the progression of many diseases (Dixon et al., 2012). Ferroptosis was observed in hrHPV-infected squamous intraepithelial lesions, indicating that it is likely to be associated with the development of CC. Cancer cells are more iron-dependent for growth and more sensitive to the iron deficiency than non-cancer cells. Ferroptosis is first reported in ovarian cancer (Wang et al., 2021a), which is another well-known gynecologic malignancy. Ferroptosis has also been reported in liver cancer (Yang et al., 2020), glioma (Zhuo et al., 2020), osteosarcoma (Liu and Wang, 2019), and renal cell carcinoma (Markowitsch et al., 2020); however, the pathogenic mechanism of ferroptosis in the progression of CC is poorly understood and needs further exploration.

## Potential mechanism of iron death and iron homeostasis in cervical cancer

Iron metabolism plays a vital role in the development of CC. A meta-analysis showed that Chinese patients with CC exhibited lower serum iron levels suggesting that higher serum iron levels may play a protective role in CC (Chen et al., 2020). The study by Braun et al., on the other hand, demonstrated that iron deficiency has profound antiviral and antiproliferative effects on HPV-positive cancer cells. The internal reason may be that tumor cells typically reprogram various cellular processes that ultimately lead to enhanced iron influx and reduced iron efflux, and iron ions enter the cells and the serum concentration of iron ions in the extracellular fluid decreases. They proposed that iron chelators, such as CPX, function as HPV inhibitors, pro-senescence agents, and pro-apoptotic agents in both normoxia and hypoxia environments. The study also demonstrated the therapeutic potential of iron chelators in cancer therapy (Braun et al., 2020). Iron is essential in many physiological processes and an essential component of hemoglobin; therefore, iron is required for oxygen transport in the body (Paul et al., 2017). Transferrin receptor 1 (TfR1) and transferrin (TF) are well-known cellular regulators of iron transport, and they control intracellular iron levels by transporting Fe^3+^ into the cell (Qian et al., 2002). Nan et al. first reported that TfR1 regulates the expression of many genes at the transcriptional and post-transcriptional levels. The results suggest that TfR1 is involved in the progression of CC by affecting the expression and alternative splicing (AS) of several genes involved in cancer-related pathways (Huang et al., 2022), but whether these pathways are related to ferroptosis has not been elucidated so far.

Although most of the intracellular iron is stored in ferritin, there is still a small cytosolic pool of weakly bound iron available for a variety of interactions with other molecules in the cell; the catalytic activity of weakly bound iron (Fe^2+^) is almost unlimited since Fe^2+^ can capture electrons to form peroxides (Dev and Babitt, 2017). These ferrous ions (Fe^2+^) are called labile iron pool (LIP) (Espósito et al., 2002; Petrat et al., 2002). Fe^2+^ is a metal ion with a high redox potential. It is the most important catalyst in the lipid peroxidation chain reaction, but it can also generate free radicals from hydrogen peroxide *via* the Fenton reaction in cells that produce a lot of ROS (Kajarabille and Latunde-Dada, 2019). Reactive oxygen species (ROS) are a group of molecules that contain partially reduced oxygen, including peroxide, superoxide, singlet oxygen, hydroxyl radicals, and free radicals. The dramatic increase of ROS in cells renders cells more prone to ferroptosis (Lin et al., 2018; Liang et al., 2019). High iron level in the cytoplasm significantly promotes ferroptosis susceptibility (Hassannia et al., 2019). Moreover, ferroptosis also participates in the regulation of iron homeostasis in the cell.

**FIGURE 1:**
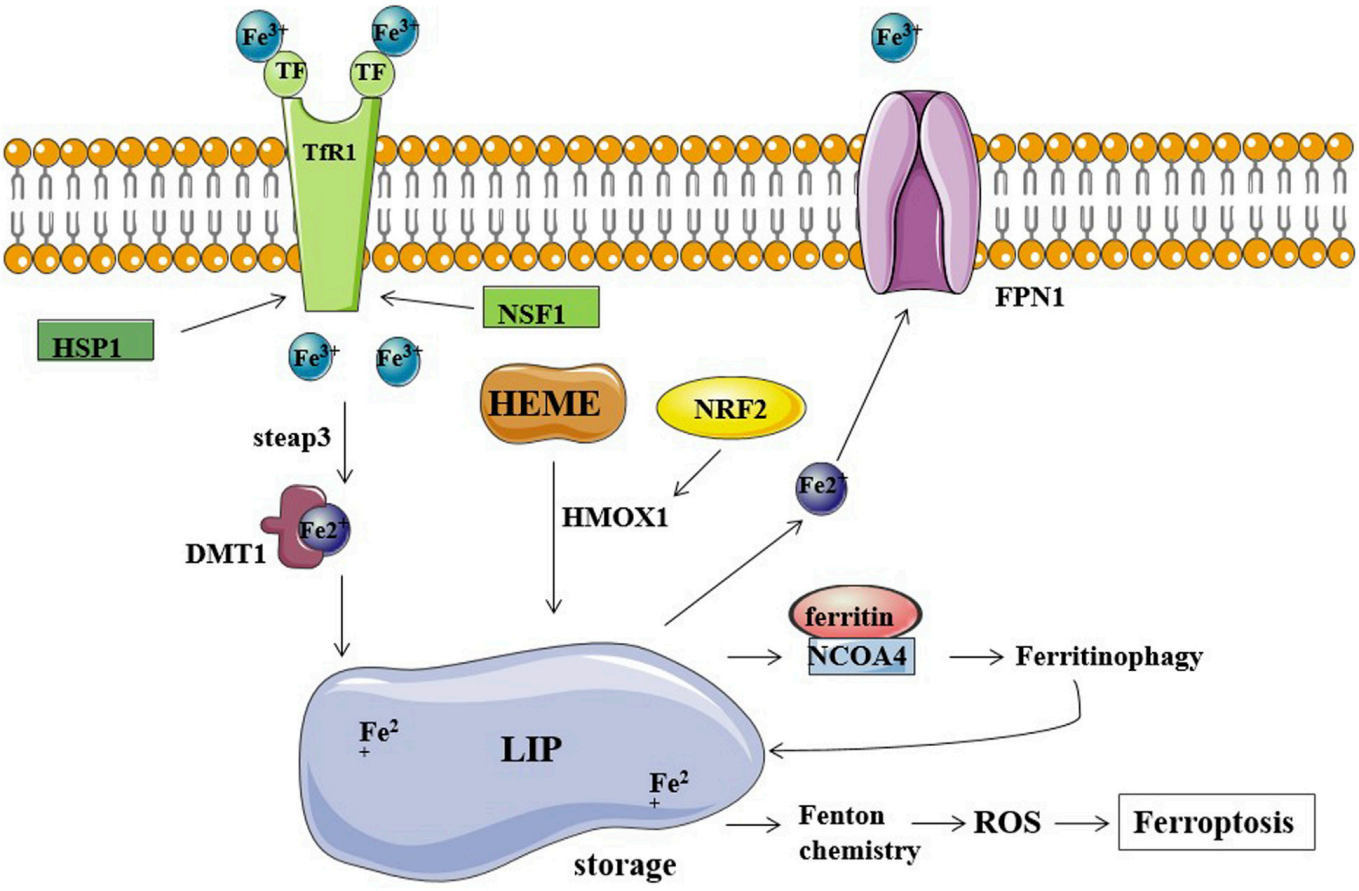
Iron metabolism and ferroptosis. Fe^2+^ storage in LIP (labile iron pool) participates in the Fenton reaction producing ROS and causing ferroptosis. TfR1, TF, and FPN1 are the major regulators of cellular iron balance on the cell membrane. TF transports Fe^3+^ into cells by TfR1-mediated endocytosis. HSP1 inhibits this process by inhibiting TfR1, while NSF1 can increase TfR1 levels. Fe^3+^ is converted to Fe^2+^ by STEAP3 and released into the cytoplasm *via* DMT1. FPN1 exports and decreases intracellular iron levels. Ferritin stores iron and limits the iron-catalyzed Fenton reaction. NCOA4 binds ferritin mediating its autophagic degradation in a process called ferritinophagy.

Genes involved in iron homeostasis (input, output, and storage of iron ions) have also been shown to modulate the sensitivity to ferroptosis. Preliminary research has been conducted on the ferroptosis regulatory factors that are involved in the release of ferric ions. The overexpression of TF and TfR1 increases iron uptake, making cells susceptible to ferroptosis; conversely, silencing TfR1 inhibited erastin-induced ferroptosis. A recent study found that heat shock protein beta-1 (HSPB1) significantly inhibits ferroptosis by inhibiting TfR1 expression and thus lowering intracellular iron levels (Li et al., 2020). Additionally, nitrogen fixation inhibitor 1 (NFS1), a cysteine desulfurase that mobilizes sulfur from cysteine to synthesize iron-sulfur clusters, is activated by simultaneously increasing TfR1 levels and decreasing ferritin levels, causing the iron starvation response, thereby sensitizing cells to ferroptosis (Alvarez et al., 2017). In addition, iron is mainly exported through ferroportin 1 (FPN1); accordingly, inhibition of FPN1 resulted in enhanced ferroptosis (Geng et al., 2018). Furthermore, the major route of iron release from ferritin is mediated by the incorporation of ferritin into the lysosomes through nuclear receptor coactivator 4 (NCOA4), and knockdown of NCOA4 decreases the ferritinophagy leading to the restricted use of free intracellular iron. NCOA4-mediated ferritin deposition is linked to neurodegeneration, as shown by Quiles et al. (Brown et al., 2019; Quiles Del Rey and Mancias, 2019). Ferritin degradation occurs through NCOA4-mediated ferritinophagy, which induces ferroptosis by releasing free iron from ferritin (Hou et al., 2016). Hemin was used as an intracellular iron source to promote ferroptosis in platelets *via* ROS-regulated proteasome activity (Naveen Kumar et al., 2019). According to one study on lung cancer, hemin causes ferroptosis in lung cancer cells while protecting normal lung cells after exposure to fractionated doses of ionizing radiation (Chen et al., 2020). Nrf2 is a transcription factor, and the activation of Nrf2 regulates iron metabolism; it reduces cellular iron uptake and limits the production of reactive oxygen species. Thus, Nrf2 inhibits ferroptosis and promotes the progression of cancer. In liver cancer cell lines, p62 can bind to Keap1 and disrupts the interaction of Keap1 with Nrf2 when exposed to compounds inducing iron toxicity. The disruption of Keap1-Nrf2 interaction by ferroptosis-inducing compounds leads to the accumulation of Nrf2, ultimately reducing cancer cell susceptibility to ferroptosis (Sun et al., 2016). In a study by Xiong et al., hypoxia upregulated KDM4A *via* H3K9me3t, which enhanced HIF1 transcription, activated HRE sequences (5′-ACGTG-3′) in TfR1, activated DMT1 promoter, and induced ferroptosis resistance in CC cells (Xiong et al., 2022) (Figure 1).

## ROS and lipid peroxidation in cervical cancer

The activation of protooncogenes in tumor cells results in the production of ROS. Furthermore, tumor cells require a large amount of nutrients and energy to maintain rapid proliferation, and tumor cells undergo metabolic reprogramming and abnormal mitochondrial function, all of which contribute to the production of more ROS. ROS have been shown to transmit proliferative signals and promote tumor development (Wang et al., 2019). Non-etheless, high levels of ROS can cause cell death, and tumor cells must increase their antioxidant activity to maintain cellular redox balance. One of the most noticeable features of ferroptosis is lipid peroxidation. Ferroptosis is a regulated cell death mechanism that results in glutathione (GSH) depletion. Ferroptosis reduces glutathione peroxidase (GPX4) activity and cellular antioxidant capacity, resulting in increased lipid peroxidation (Dixon et al., 2012; Yang et al., 2014). Sanguinarine treatment increased the generation of reactive oxygen species (ROS), and blocking ROS production inhibited the induction of both apoptosis and ferroptosis. PUFA (polyunsaturated fatty acid)-containing membrane phospholipids are vulnerable to peroxidation under intracellular environment rich in iron and reactive oxygen species. This accumulation of lipid peroxides in the cell membranes eventually destroys membrane integrity leading to ferroptosis (Stockwell et al., 2017). Ferroptosis induces specific morphological changes in mitochondria, including mitochondrial shrinkage, increased membrane density, outer membrane rupture, cristae shrinkage or disappearance, membrane potential drop, etc. Erastin-mediated inhibition of intracellular cystine causes the depletion of intracellular glutathione (GSH), eventually leading to the inactivation of GPX4 and accumulation of lipid peroxidation, inducing cell death. RSL3, a well-known inhibitor of GPX4, can also directly promote these effects, and the regulatory mechanisms of RSL3 include GSH/GPX4 axis, system Xc-, ACSL4, FSP1, etc. (Bridges et al., 2012; Yuan et al., 2016; Doll et al., 2019).

Glutathione reductase (GR), also known as Glutathione-disulfide reductase (GSR), is a key enzyme that catalyzes the reduction of GSH by glutathione disulfide (GSSG), and the inhibition of GSR results in increased ROS activity. In a previous study of ROS in the development of CC, Xia Y et al. demonstrated that the expression of GSR level was increased in human CC tissues, inhibiting the enzymatic activity of GSR and inducing cell death in CC through a ROS-dependent mechanism. Furthermore, this study demonstrates the potential of ROS as an effective antitumor modality for the treatment of CC (Fan et al., 2020). HPV oncoproteins induce oxidative stress (OS), which in turn promotes lipid peroxidation and cellular damage resulting in various types of cell death, including ferroptosis (Di Domenico et al., 2018). It has been demonstrated that the pathogenesis of HPV-mediated CC progression is linked to ferroptosis. Ferroptosis was found in low-grade cervical squamous intraepithelial lesions (SIL) with hrHPV infection in a study on the relationship between ferroptosis and HPV-induced cervical disease. Persistent ferroptosis aided in developing squamous intraepithelial lesions (SIL), which resulted in anti-ferroptotic effects. Many other studies have identified the critical role of lipid peroxidation in the progression of CC, providing us with new ideas for treating CC by targeting ferroptosis (Wang et al., 2022).

## Role of GPX4-GSH pathway in ferroptosis of cervical cancer

**FIGURE 2:**
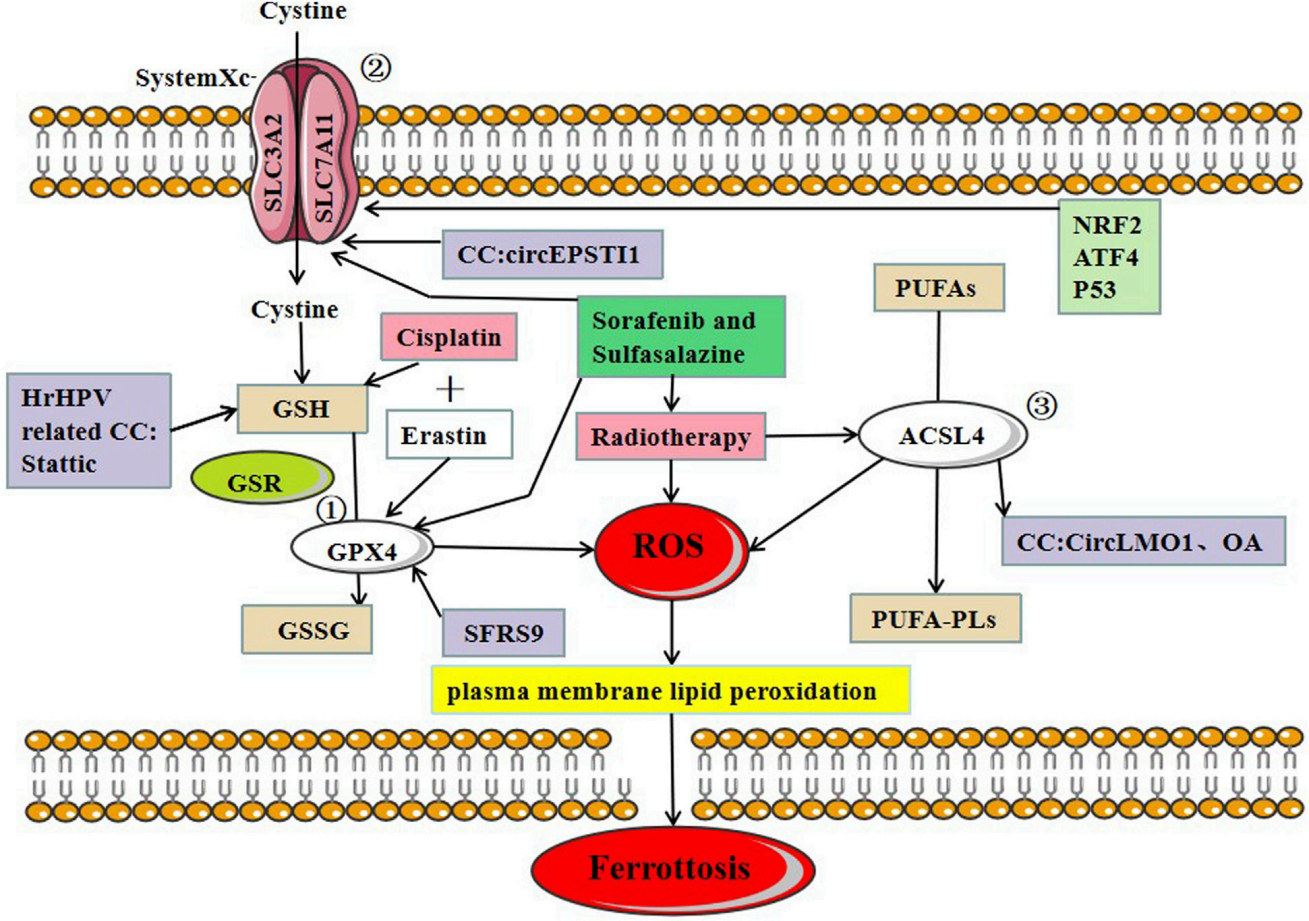
Three major regulatory mechanisms of ferroptosis in cervical cancer. ①GPX4 pathway: the SLC7A11-GSH-GPX4 (solute carrier family 7 members 11-glutathione-GPX4) signaling axis accounts for the predominant ferroptosis defense system. Cysteine serves as the rate-limiting precursor for the biosynthesis of GSH, a critical cofactor for GPX4 to detoxify lipid peroxides, ② SystemXc-: SLC7A11 is a core component of the cystine-glutamate antiporter system (system Xc-) and mediates the antiport activity of system xc− by importing extracellular cystine and exporting out intracellular glutamate, ③ ACSL4: PUFAs derived from lipid bilayers are metabolized by ACSL4 leading to the production of ROS. Recent research on ferroptosis in CC has primarily focused on these three protein complexes. The main treatments for ferroptosis-related CC are cisplatin and radiotherapy.

GPX4 plays a critical role in regulating ferroptosis, and the inhibition of GPX4 induces ferroptosis. GSH is a substrate of GPX4, and the deletion of GPX4 induces ferroptosis. Erastin inhibits ferroptosis by reducing intracellular GSH levels. RSL3 can also directly bind to GPX4 and inhibits its activity, leading to the accumulation of intracellular reactive oxygen species and ferroptosis induction (Yang and Stockwell, 2008). P53, a well-known tumor suppressor gene, can inhibit the induction of system Xc-through the classical pathway and enhances ferroptosis by inhibiting dipeptidyl-peptidase 4 through the non-canonical pathway (Jiang et al., 2015a). hrHPV protein E6 induces p53 degradation by activating hTERT (human telomerase reverse transcriptase), avoiding apoptosis and cell immortalization (Vande Pol and Klingelhutz, 2013). However, the involvement of ferroptosis in this process needs to be further verified. In cervical and bladder cancers, SFRS9 (Serine and arginine-rich splicing factor 9) has been identified as a protooncogene. SFRC9 can inhibit ferroptosis in colorectal cancer (CRC) by binding to GPX4 mRNA, and another study suggests that inhibiting SFRC9 in CRC may have therapeutic implications (Wang et al., 2021b). MiR-193a-5p targets GPX4 mRNA and reduces GPX4 expression in cervical cancer cells. Circular RNA circACAP2 increases GPX4 expression by targeting miR-193a-5p, thereby repressing ferroptosis in cervical cancer during malignant progression by miR-193a-5p/GPX4. Indeed, limited studies have been conducted to identify the role of GPX4 in inducing ferroptosis in cervical cancer cells. Further studies are required to understand the precise role of GPX4 in CC progression (Figure 2).

## Role of system Xc- in ferroptosis of cervical cancer

System Xc- is an amino acid transporter widely distributed on the cell membrane and mainly consists of SLC7A11 and SLC3A2. System Xc- transports cystine into the cytosol in exchange for the same amount of glutamate being transported out of the cell. Inhibiting the activity of system Xc- can reduce cystine absorption and decrease glutathione synthesis, eventually leading to oxidative damage and ferroptosis (Bridges et al., 2012). ATF4 (activating transcription factor 4) and NRF2 (nuclear factor erythroid 2-related factor 2) represent two major transcription factors mediating stress-induced transcription of SLC7A11, and it has been shown that SLC7A11 drives ferroptosis resistance (Ye et al., 2014). Erastin and sorafenib induce ferroptosis by inhibiting System Xc-. Multiple studies suggest that p53 downregulates the expression of SLC7A11 as demonstrated in polymorphic mutants of p53, and the mechanism of p53-mediated tumor suppression is governed by the inhibition of SLC7A11 function, conferring resistance to ferroptosis (Jiang et al., 2015b; Jennis et al., 2016; Wang et al., 2016).

A recent study of CC found that the circEPSTI1-miR-375/409–3P/515–5p/SLC7A11 axis influenced CC proliferation *via* the competing endogenous RNAs (ceRNA) mechanism and was involved in ferroptosis. Wu et al. provided experimental evidence, which revealed that circEPSTI1 might act as a new and useful prognostic and predictive biomarker for CC (Wu et al., 2021) (Figure 2).

## Role of ACSL4 in ferroptosis of cervical cancer

ACSL4 (Long-chain acyl-CoA synthetase 4) belongs to the acyl-CoA synthetase protein family, which catalyzes the covalent addition of a CoA moiety to fatty acid groups in an ATP-dependent manner. Mouse embryonic fibroblasts from ACSL4 knock-out mice undergo ferroptosis in response to RSL3 (RAS-selective lethal 3). Thus, ACSL4 plays a crucial role in iron-dependent oxidative stress (Yuan et al., 2016). Cortical neurons (Seiler et al., 2008), fibroblasts (Wortmann et al., 2013), vascular cells (Wortmann et al., 2013), T cells, and erythroid cells (Canli et al., 2016) did not survive in the absence of GPX4. ACSL4 and GPX4 double KO cells, on the other hand, survived and proliferated normally in cell culture for an extended period of time, highlighting the critical functional interaction between GPX4 and ACSL4.

In CC, CircLMO1, a newly identified circRNA, induced ferroptosis in CC cells by upregulating ACSL4 expression. Overexpression of miR-4291 or knockdown of ACSL4 reversed the effect of circLMO1 in facilitating ferroptosis, repressing proliferation, and decreasing tumor invasion of CC cells (Wu et al., 2021). Oleanolic acid (OA) promotes ACSL4-dependent ferroptosis in HeLa cells (Xiaofei et al., 2021) (Figure 2).

## Potential applications of ferroptosis in cervical cancer

Besides surgery, the first-line chemotherapy drugs for CC currently include cisplatin, paclitaxel, carboplatin, etc. The second-line chemotherapy drugs include gemcitabine, epirubicin, etc. Among various anticancer drugs, cisplatin is one of the most widely used and effective anticancer agent in the treatment of different types of solid tumors, but unfortunately, repeated platinum therapy after tumor recurrence is often ineffective. Cisplatin resistance may develop through the following molecular mechanisms: increased DNA repair capacity, altered cellular aggregation of the drug, and cytoplasmic inactivation of the drugs (Richon et al., 1987; Ferry et al., 2000; Amable, 2016). These cell death mechanisms are involved in apoptosis, but whether ferroptosis occurs through similar mechanisms is unclear. Ferroptosis is a novel cell death mechanism that differs greatly from traditional apoptosis; thus, targeting ferroptosis may be an effective therapeutic strategy for overcoming tumor resistance to cisplatin. Given that elastin induces ferroptosis, combining elastin (GSH inhibitor) with cisplatin (genotoxic agent) may have a synergistic effect on cancer therapy (Figure 2). Cisplatin combined with elastin showed a significant additive effect on antitumor activity in human ovarian cancer, head, and neck cancer (HNC), lung carcinoma (A549), and colon carcinoma (HCT116) cell lines, according to Guo et al. and Roh et al. (Roh et al., 2016; Guo et al., 2018). In oxaliplatin-resistant human cervical cancer cell lines, a combination treatment of iron chelator desferal (DFO) and oxaliplatin can overcome oxaliplatin resistance (Chen et al., 2016); a combination of low-concentration of PTX and RSL3 synergistically inhibits tumor cell growth by inducing ferroptosis (Ye et al., 2019); both *in vivo* and *in vitro* experiments confirmed that a combination of gemcitabine and erastin can inhibit the HSPA5-GPX4 signaling pathway and displays a synergistic antitumor effect on pancreatic cancer cells. A low concentration of elastin enhances the sensitivity of HL-60 cells to cytarabine and doxorubicin (Zhu et al., 2017) (Table 1).

**TABLE 1:** Overview of ferroptosis-associated chemotherapeutic agents in CC.

Chemotherapeutic agents	*In vitro*	*In vivo*	Inhibitors and inducers of ferroptosis	Effect
Oxaliplatin Chen et al. (2016)	Oxaliplatin-resistant human cervical cancer cell line, S3	SiHa and S3 tumor xenograft mouse models Chen et al. (2016)	Deferoxamine/DFO	The synergistic killing effect
Cisplatin Roh et al. (2016); Guo et al. (2018)	Human ovarian cancer cell line (A2780 and its CDDP-resistant variant A2780DDP), HNC cell lines (AMC-HN3R, -HN4R, and -HN9R), A549, HCT116 cells	HN9-cisR mouse models Roh et al. (2016)	Erastin	The synergistic killing effect, enhancing the anticancer activity
Paclitaxel/PTX Ye et al. (2019)	mtp53 HPSCC	None	RSL3	Enhance the anticancer activity
Gemcitabine Zhu et al. (2017)	PANC1, CFPAC1, MiaPaCa2, Panc2.03, and Panc02 cells	PANC1 nude mouse model	Erastin	Enhance sensitivity
Cytarabine, Doxorubicin Zhu et al. (2017)	HL-60 cells	None	Erastin	Enhance the anticancer activity

Radiation therapy is another effective modality for the treatment of CC, but some patients develop resistance to radiation therapy. Various ferroptosis inducers, such as sorafenib and sulfasalazine, can act as radiosensitizers by inhibiting SLC7A11 and GPX4 activity. Lei et al. (Lei et al., 2020) found that radiotherapy can induce tumor cells to produce a large amount of lipid ROS and ACSL4 leading to the increased accumulation of lipid peroxides and increased occurrence of ferroptosis (Figure 2). The study further reported the activity of SLC7A11 and GPX4 in combination with ferroptosis inducers to cervical cancer *in vitro* and *in vivo* experiments, and found that tumor cells were considerably more sensitive to radiotherapy. The study also suggested that suitable ferroptosis inducers can serve as effective radiosensitizers to improve the efficacy of radiotherapy on tumor cells.

## Conclusion

In other studies investigating the role of ferroptosis in cervical cancer, Wang et al. show that Cdc25A (cell division cycle 25) upregulates ErbB2 (epidermal growth factor receptor) level through dephosphorylation of PKM2, thereby inhibiting autophagy-dependent ferroptosis in CC cells (Wang et al., 2021c). Zhao et al. demonstrate that propofol and paclitaxel exert synergistic anticancer effects on cervical cancer cells (Zhao et al., 2022). Qi et al. established a four-gene (TFRC. ACACA, SQLE, and PHKG2) prognostic signature based on ferroptosis related genes (FRGs), providing new targets for CC involving ferroptosis (Qi et al., 2021). Xing et al. discovered eight ferroptosis- and immune-related differentially expressed genes (FI-DEGs) and developed a risk assessment model to predict outcomes in CESC patients (Cervical squamous cell carcinoma). Therefore, these eight genes have the potential to be prognostic and predictive biomarkers for cancer. Indeed, more research is needed to confirm the findings in the field of CC (Xing et al., 2021). Jiang et al. established a new predictive model that integrated 7 lncRNAs related to ferroptosis through analysis, which improve the predictive value and guided personalized treatment in patients with CC (Jiang et al., 2022). In the study by Zou et al., the ferroptosis-related gene PTGS2 turned out to be a key prognostic gene for an early-stage CC model associated with the immune microenvironment (Zou et al., 2022). Li et al. established a ferroptosis score (FerroScore) that was used to predict the sensitivity to chemotherapy and responses to immunotherapy in patients with CC. All these methods have a potential application for ferroptosis in CC.

Cancer incidence has been rising in recent years. According to current research, the ability of cells to avoid apoptosis is the primary cause of tumor resistance to treatment. Because ferroptosis differs from apoptosis, it provides a novel therapeutic option for cancer treatment. Due to the important roles of iron in cellular metabolism and increased oxidative stress in cervical cancer, it is worth exploring whether ferroptosis plays an important role in the pathogenesis of cervical cancer. In addition to the above-mentioned proteins and non-coding RNAs, several other compounds and proteins also play important roles in the occurrence of ferroptosis, regulating the biological characteristics of CC cells. HPV infection is closely related to the early diagnosis of CC, and HPV infection is also associated with oxidative stress, which is a key process promoting cell death by ferroptosis. Therefore, understanding the underlying mechanism of ferroptosis in the progression of CC may have implications for early diagnosis of CC.

In addition, the present study demonstrates that ferroptosis significantly affects the sensitization of tumor cells to chemoradiotherapy. Depending on the clinical status of CC, many patients with CC are resistant to traditional treatments. Therefore, as a newly discovered cell death mechanism, ferroptosis has excellent research value in antitumor therapy. However, antitumor therapy for ferroptosis faces numerous challenges. Several studies on the mechanism of ferroptosis in CC are currently being conducted, but the clinical application of the ferroptotic pathway in cancer therapy is very limited. Ferroptosis research in CC is currently ongoing, and the future application prospects of ferroptosis are limitless.
